# Recent advances in veterinary applications of structural vaccinology

**DOI:** 10.1016/j.coviro.2018.02.006

**Published:** 2018-04

**Authors:** Bryan Charleston, Simon P Graham

**Affiliations:** The Pirbright Institute, Ash Road, Pirbright, Guildford GU24 0NF, Surrey, United Kingdom

## Abstract

The deployment of effective veterinary vaccines has had a major impact on improving food security and consequently human health. Effective vaccines were essential for the global eradication of Rinderpest and the control and eradication of foot-and-mouth disease in some regions of the world. Effective vaccines also underpin the development of modern intensive food production systems such as poultry and aquaculture. However, for some high consequence diseases there are still significant challenges to develop effective vaccines. There is a strong track record in veterinary medicine of early adoption of new technologies to produce vaccines. Here we provide examples of new technologies to interrogate B cell responses and using structural biology to improve antigens.

## Highlights

• Applying structure-based vaccine design as a route to induce broadly protective immunity to viral infections.
• Structural vaccinology as an innovative tool to develop stable antigens that induce enhanced immunity against viruses.

**Current Opinion in Virology** 2018, **29**:33–38

This review comes from a themed issue on **Preventive and therapeutic vaccines**

Edited by **Marc van Regenmortel**

For a complete overview see the Issue and the Editorial

Available online 16th March 2018

**https://doi.org/10.1016/j.coviro.2018.02.006**

1879-6257/© 2018 The Authors. Published by Elsevier B.V. This is an open access article under the CC BY license (http://creativecommons.org/licenses/by/4.0/).

Protective antibody responses are an essential component of the responses induced by effective vaccines for a number of pathogens. Antibodies can recognise short peptides that contain key residues that form the epitope, but these peptides might lack additional residues that are outside of the linear epitope, so they do not represent the structures recognised by antibodies in immunised or infected animals. Currently, quantitative rules linking sequence, structure, immunogenicity, and protection are still lacking. The induction of robust protective immune responses in livestock species still relies on the presentation of antibody epitopes in the context of the whole pathogen or individual proteins. Therefore, the immediate application of structural vaccinology to enhance protective antibody responses will be the engineering of proteins to maintain epitopes rather than the use of isolated epitopes [1]. The use of structural information in vaccine design will allow proteins to be modified to produce more stable vaccine antigens to ensure the structure of key epitopes are maintained during vaccine production, distribution and application. There are two examples of the application of structural vaccinology for vaccine design that have been validated by *in vivo* proof of concept studies in the livestock target species, these are FMD virus like particle (VLP) vaccines and bovine RSV (bRSV) fusion (F) protein.

## Structure-based vaccine design as a route to broadly protective PRRS vaccines

PRRS continues to be the key economically important pig disease worldwide. The causative PRRS viruses (PRRSV) are rapidly evolving, as most dramatically illustrated by the emergence of highly pathogenic variants in Southeast Asia and Eastern Europe [2, 3, 4]. Thus, more efficacious control strategies are urgently sought. Current vaccines can confer protection from disease but show variable efficacy against challenge with heterologous strains [5, 6, 7, 8, 9, 10, 11, 12]. A variety of experimental subunit approaches have been evaluated as potential next-generation vaccines but have at best conferred only limited protection [13, 14, 15, 16, 17, 18]. The limitations of both existing and experimental vaccines support the proposition that a new approach is required to design immunogens capable of providing broad protection against this hypervariable pathogen. Neutralising antibodies (nAbs) can provide immunity against PRRSV as demonstrated by a dose-dependent protective effect conferred by passive transfer of homologous PRRSV-neutralising sera [19]. However, the PRRSV-specific Ab responses measurable from 7 days post-infection is non-neutralising, and nAb responses are often not observed until at least four weeks post-infection and titres, when measurable, are often lower than those elicited by other viral infections [20, 21]. Collectively, these observations suggest that PRRSV has evolved strategies to modulate the B cell response to evade the induction of protective nAbs via the glycan shielding of neutralising epitopes and/or the promotion of responses against non-neutralising decoy epitopes [22]. These data also suggest that vaccination strategies that induce high-titre nAbs would be efficacious. The development of such a strategy would benefit from an improved understanding of the neutralising epitopes on PRRSV that confer protection. Since a lack of cross-protection is a major constraint in the development of PRRSV vaccines, the identification of conserved epitopes is of paramount importance.

Linear nAb epitopes have been identified on GP2, GP3 and GP4 [23, 24] of PRRSV-1 and GP5 of both PRRSV-1 and PRRSV-2 [25, 26, 27]. The complexity of the nAb response to PRRSV and the limitation of current understanding was illustrated by recent studies investigating the cross-neutralisation of field strains [28, 29]. Evaluation of the neutralisation of PRRSV-1 isolates by a panel of hyperimmune sera revealed significant differences in the sensitivity of PRRSV strains to neutralisation; however, no correlation was observed with known linear nAb epitopes or N-linked glycosylation sites [29]. Interestingly, 10% of sera exhibited significant neutralising activity against all isolates, suggesting that these sera contain nAb specific for conserved epitopes that may be poorly exposed and consequently weakly immunogenic in most strains. Whilst it may be suggested that differences in residues outside the described linear epitopes altered accessibility, an alternative explanation is the presence of as-yet-unidentified conformational nAb epitopes. In support of this, a study of PRRSV-2 infected pigs identified a single animal with a broadly nAb response that even neutralised PRRSV-1 [30^•^]. The deletion of a single non-conserved amino acid in the M protein conferred resistance to this response, although it remains to be determined whether this mutation is altering a conformational epitope or blocking access to a linear epitope. Evidence for the natural occurrence of broadly nAbs was presented in a recent study of two US sow herds with multiple exposures to PRRSV that revealed sera capable of neutralisation of strains spanning both PRRSV species [31^•^]. The identification of nAb epitopes and particularly the conserved viral structures recognised by these broadly nAbs would lay the solid basis on which to rationally develop a much-needed second generation vaccine to prevent and control PRRS.

Identification of the epitopes recognised by broadly nAbs is an area of intense recent research in the context of a number of highly variable human viruses [32, 33, 34, 35]. Central to this effort are methods to generate and analyse the specificity of naturally occurring monoclonal antibodies (mAbs). Recent advances in methodologies to analyse antigen-specific B cells and their immunoglobulin genes are now providing large numbers of human mAbs for application both as therapeutics and in the design of novel immunogens [36]. We are pursuing two complementary approaches to isolate broadly neutralising mAbs from a cohort of pigs hyper-immunised by sequential challenge infections with heterologous PRRSV strains. The first approach involves the use of a retroviral vector to constitutively express the B cell lymphoma-6 (Bcl-6) transcription factor and the anti-apoptotic Bcl-2-like protein 1 (Bcl-xL) in memory B cells [37^••^]. With co-stimulation, transduced cells proliferate, secrete their Abs and retain surface immunoglobulin expression. These cells are therefore amenable to both enrichment/cloning by antigen-baiting and direct analysis of Ab specificity in cell culture supernatants. This approach has been successfully deployed to isolate human mAbs capable of broadly neutralising human parechovirus [38], RSV [37^••^] and influenza A virus [39], to eventually identify epitopes as vaccine targets. Significantly, this approach has been used to successfully immortalise B cells from rabbits, mice, llamas and non-human primates [37••, 40]. The second approach, which builds on our experiences in isolating FMDV-neutralising bovine mAbs, is using high-throughput sequencing of immunoglobulin genes from plasma B cells. Bioinformatic analysis will identify over-represented clonal families that will be expressed as recombinant mAbs using an established high-throughput protein expression pipeline. Cross-neutralising mAbs will be identified by *in vitro* screening epitopes elucidated by cryo-EM structural analysis of mAb complexed to purified PRRSV virions. We hope that the isolation of these mAbs will contribute to both our understanding of the nAb response to PRRSV and allow epitopes to be resolved that may be engineered as immunogens to induce cross-protective immunity.

## Structural vaccinology approaches to induce enhanced immune responses against FMDV and RSV in cattle

**Figure 1:**
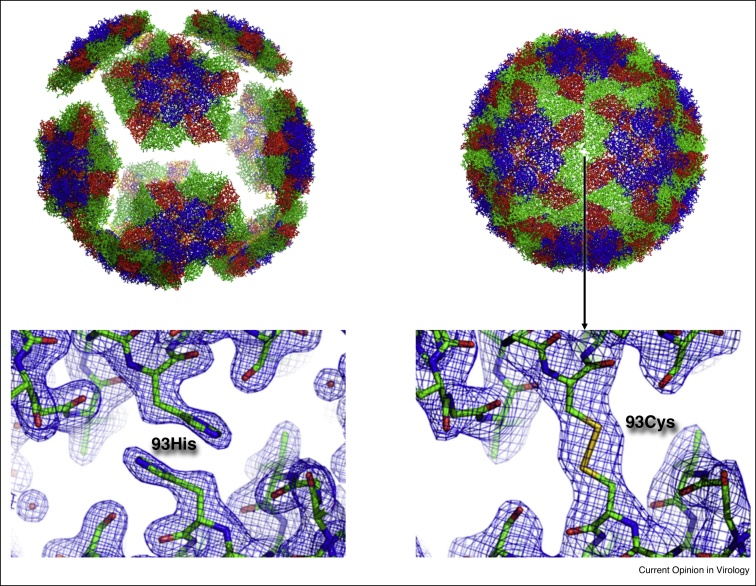
Precise changes to the structure of viral proteins can significantly enhance their antigenic properties. The example shown on the left is a foot-and-mouth disease virus capsid that readily dissociates into pentamers that will not induce protective immune responses. However, if point mutation is introduced at the boundary of the pentamers to form a new covalent bond, compare left and right lower structures, the intact capsids are stabilised and can survive heat or acid treatment and induce protective responses.

FMD is endemic in large parts of South America, Africa, The Middle East and Asia and is, globally, the most economically important infectious disease of livestock, affecting cattle, pigs, sheep, goats and other artiodactyl species (Figure 1). In endemic countries, FMD not only affects national and international trade, but also impacts on the whole livestock industry with damaging consequences for local farmers and, invariably, loss of income. Livestock health is clearly linked to human health and prosperity, hunger, malnutrition, and poor health are widespread and stubborn development challenges. Routine vaccination programmes are employed in the endemic regions of the world but regular immunisation is required. Improved vaccines, in terms of stability and protection against emerging FMDV, are essential for disease control and to establish and maintain FMD-free status in many regions of the world. The current FMD vaccines are inactivated whole virus preparations which contain an adjuvant to enhance the immune response. There are 7 FMDV serotypes: O, A, Asia1, C and three strains circulating predominately in sub-Saharan Africa, SAT (South Africa Territories) 1, 2 and 3.

There is currently a massive shortfall in the availability of vaccines, most strikingly in Africa. There is a significant impediment to building new high disease containment facilities to increase the production of conventional killed virus vaccines because of the high initial and on-going costs. A particular difficulty for FMDV is that effective vaccination requires the presence of intact inactivated virus or VLPs. Individual proteins or peptides have proven to be insufficiently immunogenic for use as vaccine antigen.

We are aiming to address these challenges. Detailed knowledge of the atomic structure of the different serotypes and strains of the virus has resulted in significant advances to produce recombinant FMD VLPs as effective vaccines that can be manufactured in conventional low containment facilities. Novel methods have been developed to design specific changes in the VLP to allow them to remain intact during production, formulation, storage and transport and so remain immunogenic.

Initial studies have reported a stable disulphide bond at the icosahedral two-fold axis between adjacent pentamers in the A22 virus, thus allowing the production of thermostable and pH-stable recombinant empty particles [41^••^]. However, there is not one simple solution to stabilise all serotypes and strains of FMDV. Consequently, a highly restrained atomistic molecular dynamics simulation *in silico* method has been developed to focus on the appropriate part of the complete virion to efficiently and reliably calculate the changes in binding free energy for interactions between adjacent pentamers within the FMDV capsid. Single-residue mutations were identified that resulted in enhanced stability by hydrophobic stacking of aromatic side chains of residues introduced at position 93 of the FMDV capsid protein VP2 at the two-fold axis between adjacent pentamers.

*In vitro* assays were used to determine that the noncovalent stabilisation particles, using hydrophobic stacking, produced similar stability to covalently cross-linked A22 particles. The potential value of this increased stabilisation was demonstrated *in vivo*. After storage for 1 or 6 months at 4 °C, stabilised particles formulated in oil adjuvant produced substantially higher virus neutralising antibody titres in guinea pigs than did wild-type particles [42••, 43].

Bovine respiratory syncytial virus (bRSV) is responsible for the majority of respiratory disease in cattle annually resulting in considerable morbidity and losses [44]. Human RSV (hRSV) is responsible for over 3 million hospitalisations for severe respiratory illness in young children and the elderly each year and for which no licensed vaccine is available [45]. Although several licensed vaccines are available for bRSV, none are fully effective, inactivated bRSV vaccines may enhance disease and have reduced efficacy in the presence of maternal antibodies [46, 47] and live vaccines can exacerbate bRSV disease [48]. Recombinant subunit-based vaccines have the potential to induce protective antibody responses with the benefits of ease of manufacture and long-term stability.

The most potently neutralising RSV antibodies identified thus far target the pre-fusion (pre-F) form of the RSV F glycoprotein [49, 50]. The pre-F form of RSV F is metastable and spontaneously undergoes structural rearrangements to the post-fusion (post-F) form, which no longer presents epitopes for many potently neutralising antibodies. Structure-based design has been applied to engineer thermostable versions of the pre-F hRSV F glycoprotein by the addition of new disulphide covalent bonds and by cavity filling through the introduction of residues with hydrophobic side chains. These mutations stabilised the pre-F conformation and retained the epitopes to induce highly potent neutralising antibodies [51••, 52]. The F glycoprotein of bRSV has greater than 80% sequence identity with that of hRSV2 so similar mutations were transferred to bRSV to create bRSV F trimer immunogens stabilised in the pre-F state. Inoculation of calves with the stabilised pre-F bRSV trimers resulted in complete microbiological and clinical protection to heterologous bRSV challenge. The high titre protective response elicited by the prefusion-stabilised F in calves suggests similar highly protective immune responses can be generated in humans immunised with similarly stabilised pre-F proteins [53^••^].

## Conclusions

Recent developments in veterinary vaccinology have paralleled the scientific advances and understanding in areas of immunology, molecular and structural biology of viruses, virus–host interactions and molecular pathogenesis of important infectious viral diseases of different livestock species. We draw attention to the value of applying structure-based vaccine designs to induce broadly-protective immune responses in the fight against viral infections such as PRRS. The significance of taking advantage of structural insights to develop stable protective antigens to induce enhanced immune responses against diseases such as FMD and RSV, will also be valuable as a ‘one health’ concept in protecting animals and humans through innovative vaccines.

## References and recommended reading

Papers of particular interest, published within the period of review, have been highlighted as:

• of special interest
•• of outstanding interest
